# Proteostasis and protein quality control in chloroplasts: mechanisms and novel insights related to protein mislocalization

**DOI:** 10.1093/jxb/eraf182

**Published:** 2025-05-02

**Authors:** Nicolaj Jeran, Maxime Mercier, Paolo Pesaresi, Luca Tadini

**Affiliations:** Dipartimento di Bioscienze, Università degli Studi di Milano, Milano, Italy; Dipartimento di Bioscienze, Università degli Studi di Milano, Milano, Italy; Dipartimento di Bioscienze, Università degli Studi di Milano, Milano, Italy; Dipartimento di Bioscienze, Università degli Studi di Milano, Milano, Italy; University of Hohenheim, Germany

**Keywords:** Chaperone and proteases, chloroplast biology, chloroplast degradation, intracellular protein trafficking, protein homeostasis, signalling

## Abstract

The dynamic rearrangement of the proteome and the maintenance of protein homeostasis (proteostasis) are crucial for the proper development and functionality of cellular compartments. Disruptions in proteostasis can severely compromise cellular health, leading to the accumulation of misfolded or mislocalized proteins prone to forming toxic aggregates. In chloroplasts, proteostasis presents unique challenges due to their endosymbiotic origin, complex subcompartmentalization, and constant exposure to reactive oxygen species (ROS) generated during photosynthesis. To counteract these challenges, chloroplasts employ sophisticated quality control systems, including chaperones, proteases, and protein degradation pathways such as ubiquitination and autophagy-related mechanisms. Additionally, cytosolic systems play a crucial role in regulating nuclear-encoded, plastid-targeted proteins, ensuring their proper delivery or degradation when defective. Within chloroplasts, specialized proteases, chaperones, and the chloroplast unfolded protein response (cpUPR) oversee protein quality and resolve aggregates to maintain functional integrity. This review critically examines mechanisms governing intracellular trafficking of plastid-targeted proteins, emphasizing key pathways and regulatory bottlenecks that, when disrupted, lead to the accumulation of mislocalized or orphan proteins. Particular focus is given to the signalling pathways that coordinate cytosolic and plastid effectors to sustain chloroplast function. Furthermore, we propose a novel role for PSBO, a subunit of the oxygen evolving complex associated with PSII, in linking proplastid-to-chloroplast differentiation with plastid quality control.

## Teaser

The trafficking of plastid-targeted proteins is discussed, focusing on regulatory bottlenecks and mislocalization. PSBO, a subunit of PSII, is suggested to link proplastid-to-chloroplast differentiation with plastid quality control.

## Introduction

Proteins are the fundamental building blocks and functional effectors of cellular structures and processes. The ability to dynamically regulate protein accumulation and localization is crucial for the development and adaptation of cellular organelles and cells. Protein homeostasis, or proteostasis, encompasses a complex network of processes that govern the synthesis, folding, assembly, localization, turnover, and maintenance of proteins (Balch *et al.*, 2008). To ensure proper cellular function, the proteome of each cellular compartment must be continuously under maintenance, ensuring that proteins accumulate in correct amounts, fold properly, localize appropriately, and assemble into functional complexes.

Disruptions in proteostasis mechanisms, whether due to environmental stressors or defects in the molecular machinery, can lead to the accumulation of misfolded, mislocalized, or structurally compromised proteins. Such defective proteins often exhibit functional impairment and a propensity to form toxic aggregates, making it essential to prevent their accumulation. This is particularly critical for two classes of aberrant proteins: (i) mislocalized proteins, which are delivered to incorrect compartments; and (ii) orphan proteins, which are out of stoichiometric balance or fail to interact with their intended partners. To counteract these challenges, cells employ sophisticated quality control mechanisms operating at multiple levels, from individual proteins to entire cellular compartments (Juszkiewicz and Hegde, 2018; Kong *et al.*, 2021), aimed at preventing damage, correcting defects, and ultimately degrading irreparable proteins. Chloroplast proteostasis presents additional complexity due to several factors: (i) the chloroplast proteome comprises both nuclear- and plastid-encoded proteins; (ii) the organelle is subcompartmentalized by the thylakoid membrane system; (iii) photosynthesis generates reactive oxygen species (ROS), which pose a threat to proteostasis; and (iv) the evolutionary origin of the chloroplast has integrated both prokaryotic and eukaryotic components (Howe *et al.*, 2008; Jarvis and López-Juez, 2013; Jan *et al.*, 2022; Li and Kim, 2022).

Chloroplast proteostasis begins in the cytosol, the primary site of plastid-targeted protein synthesis. Cytosolic quality control systems, including chaperones and the ubiquitin–proteasome system (UPS), regulate plastid protein precursors, ensuring their proper delivery to the plastid import machinery or targeting them for degradation if defective (Kessler, 2012; Thomson *et al.*, 2020; Sun and Jarvis, 2023). Additionally, in response to developmental cues or environmental stress, cytoplasmic pathways such as vacuole-mediated autophagy can facilitate the degradation of entire chloroplasts when necessary (Woo *et al.*, 2018; Woodson, 2019). Within the chloroplast itself, a sophisticated network of proteases and chaperones ensures the import, maturation, suborganellar localization, and maintenance of plastid proteins (van Wijk, 2015, 2024; Llamas and Pulido, 2022). These cytosolic and plastid proteostasis mechanisms are tightly regulated by various signalling pathways, including feedback from the proteome and its proteolytic products (Kaushik and Cuervo, 2015; Read and Schröder, 2021). This review examines the intricate processes sustaining plastid proteostasis and, consequently, chloroplast homeostasis. Particular focus is given to the signalling pathways governing these mechanisms and the coordination between cytosolic and plastid effectors in maintaining chloroplast functionality.

## Regulation and cytosolic control of plastid precursor proteins

**Fig. 1.**
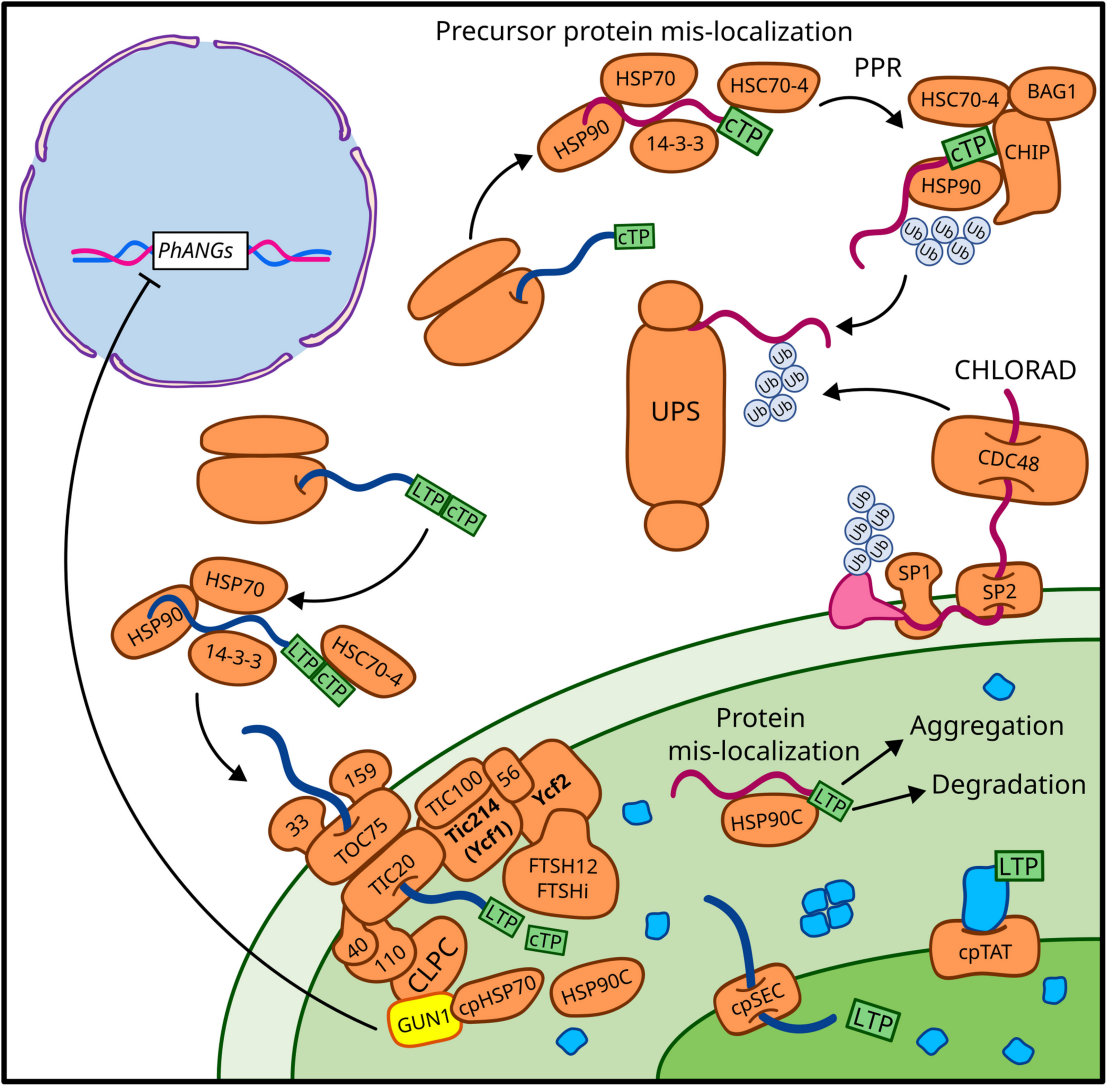
Intracellular trafficking of plastid precursor proteins. Key pathways and regulatory bottlenecks that, when disrupted, lead to the accumulation of mislocalized or orphan proteins are displayed. Nuclear-encoded plastid precursor proteins, such as those associated with *Photosynthesis-Associated Nuclear Genes* (*PhANGs*), are synthesized by cytosolic ribosomes. These proteins contain an N-terminal chloroplast transit peptide (cTP) that directs their import into the chloroplast. For proteins destined for the chloroplast lumen, an additional lumenal transit peptide (LTP) is required. Proper intracellular trafficking ensures that proteins reach their correct subcellular destinations, where they assemble into functional protein complexes (in blue). The plastid precursor response (PPR) plays a crucial role in managing plastid precursor proteins through a network of chaperones (e.g. HSC70-4), ubiquitin ligases (e.g. CHIP), the cytosolic ubiquitin–proteasome system (UPS), and, in the case of chloroplast outer envelope proteins, the CHLORAD system. Plastid-encoded protein subunits Ycf1 and Ycf2 are indicated in bold, while signalling mediators, such as GUN1-dependent retrograde signalling, are highlighted in yellow.

Although the majority of the cellular proteome is synthesized by cytosolic ribosomes, approximately two-thirds of the proteins synthesized in the cytosol are transported from there to various cellular compartments (Hegde and Zavodszky, 2019). Intracellular trafficking depends on signal peptides and localization machinery that are crucial for directing proteins to their correct destinations. This coordination is essential for cellular development and proteome homeostasis. Nuclear-encoded plastid-localized proteins are synthesized as precursor proteins and delivered to the chloroplast surface by heat shock protein chaperones, such as HSP70 and HSP90 (Fig. 1; Flores-Pérez and Jarvis, 2013). In addition to outer envelope membrane (OEM) proteins that rely on intrinsic hydrophobic transmembrane domains for targeting (Kim *et al.*, 2019), protein import into plastids requires chloroplast transit peptides (cTPs), intrinsically disordered regions located at the N-terminus of pre-proteins (Bruce, 2000). cTPs are recognized by chaperones, and translocon complexes at the outer (TOC) and inner (TIC) chloroplast envelope facilitate the translocation of precursors as unfolded entities (Lee *et al.*, 2013). These cytosolic unfolded precursors are prone to aggregation if not promptly imported into plastids (Baker and Bernardini, 2021; Rochaix, 2022), and multiple factors, such as environmental stresses, chemical treatments, or genetic mutations, can reduce import rates, leading to their accumulation in the cytosol (Dutta *et al.*, 2009; Ling and Jarvis, 2015; Tadini *et al.*, 2020).

The accumulation of unimported plastid precursors is managed by the cytosolic protein quality control (PQC) system, which monitors both newly synthesized and pre-existing proteins in a continuous process (Baker and Bernardini, 2021). The cytosolic PQC ststem comprises a network of chaperones that assist with *de novo* folding or the refolding of client proteins, as well as the UPS, which tags and degrades unfolded or misfolded proteins (Fig. 1; Saibil, 2013; Amm *et al*., 2014; Baker and Bernardini, 2021). Being in an unfolded state, plastid precursor proteins mislocalized in the cytosol activate specific PQC pathways to address malfunctioning chloroplast protein import. For example, the *Arabidopsis thaliana ppi2* mutant, which lacks the TOC159 import receptor, exhibits defective plastid protein import, resulting in altered proplastid-to-chloroplast differentiation (Bauer *et al.*, 2000). However, plastid precursor proteins do not accumulate in the cytosol of *ppi2* cells, as the UPS clears the cytosol by activating the expression of protein folding- and protein degradation-related genes, triggering a plastid-precursor-specific response (PPR) (Bauer *et al.*, 2000; Lee *et al.*, 2010). The main players involved in the PPR are the cytosolic HSC70-4 chaperone and the E3 ligase CHIP (Fig. 1). When plastid precursors accumulate in the cytosol, the expression of both HSC70-4 and CHIP genes is induced, along with genes encoding proteasome subunits. HSC70-4 specifically recognizes sequence motifs in cTPs of the precursors, while CHIP facilitates the ubiquitination of client precursors by directly interacting with the C-terminal region of HSC70-4, marking them for degradation via the UPS (Lee *et al.*, 2010). Additionally, BAG1 was identified among the transcripts induced in *ppi2* cells (Lee *et al.*, 2010). In mammalian cells, BAG (Bcl-2-associated athanogene) proteins function as regulators of HSP70. Two out of six human BAGs possess a ubiquitin-like domain at the N-terminus, allowing them to interact with the proteasome (Alberti *et al.*, 2003). In Arabidopsis, BAG1, along with HSC70-4, contributes to the proteasome-dependent degradation of unimported plastid precursors (Fig. 1; Lee *et al.*, 2016). The precise recognition of distinct sequence motifs in cTPs by HSC70-4 ensures the high specificity of PPR, as demonstrated by its inability to alleviate the artificially induced cytosolic accumulation of mitochondrial precursor proteins, as well as vacuole-targeted or cytosolic proteins (Lee *et al.*, 2010). A similar mechanism for handling mislocalized mitochondrial proteins has been described in yeast, where cytosolic factors detect and target precursor accumulation caused by defective import machinery (Wrobel *et al.*, 2015). Additionally, cytosolic condensate bodies formed through phase separation offer a means to sequester misfolded proteins. This mechanism, however, is not specific to plastid precursor proteins but also involves plastid-located proteins, such as the ankyrin-repeat proteins STT1 and STT2 (Fan *et al.*, 2024). This process is extensively studied in animal systems for its implications in diseases, and is recently emerging in plants (Wang *et al.*, 2021; Liu *et al.*, 2024).

Among PQC pathways, chloroplast-associated protein degradation (CHLORAD) specifically targets, extracts, and degrades chloroplast OEM proteins within the cytosol (Fig. 1; Thomson *et al.*, 2020). CHLORAD depends on the SP1 E3 ligase to mediate the ubiquitination of TOC proteins, while the channel protein SP2, together with the cytosolic chaperone CDC48, delivers targeted proteins to the 26S proteasome for degradation (Ling *et al.*, 2012, 2019). In this context, the transmembrane E3 ligase SP1 plays an important regulatory role in chloroplast protein import in response to developmental cues and environmental fluctuations (Ling *et al.*, 2012; Ling and Jarvis, 2015). SP1 specifically mediates the degradation of TOC components, contributing to the remodelling of the chloroplast proteome by regulating the composition and function of the chloroplast import apparatus. Aspects related to the regulation of chloroplast protein import will be further discussed in the upcoming paragraphs. SP1 also plays a role in tomato fruit ripening, as it is essential for the chloroplast-to-chromoplast transition. This underscores the significant impact of the CHLORAD-mediated protein import machinery on plastid proteome composition (Ling *et al.*, 2021). Additionally, the removal of SP1 significantly impairs seedling viability under abiotic stresses such as high salinity, osmotic shock, and methyl viologen treatments, while its overexpression improves stress tolerance (Ling and Jarvis, 2015). TOC components can also be selectively targeted for autophagic degradation by NBR1, influencing chloroplast tolerance to UV irradiation and heat stress (Wan *et al.*, 2023). Besides ubiquitin-dependent systems, the small ubiquitin-like modifier (SUMO) system negatively regulates chloroplast OEM protein accumulation and may serve as an alternative mechanism to SP1 for directing substrates to the proteasome (Watson *et al.*, 2021).

Recent studies proposed that the UPS system can target chloroplast-located proteins through the CHLORAD system, raising an intense debate on the subject (Li *et al.*, 2022; Sun *et al.*, 2022; Jarvis *et al.*, 2024; van Wijk and Adam, 2024). Disruption of either CHLORAD or proteasomes has been shown to result in the accumulation of polyubiquitinated plastid proteins within chloroplasts, including the plastid-encoded Rubisco large subunit (RbcL) and ATP synthase beta subunit (AtpB) (Li *et al.*, 2022). However, it remains unclear whether plastid proteins undergo ubiquitination within the chloroplast, given the absence of canonical ubiquitin-activating enzymes, or if misfolded proteins are transiently ‘retro-translocated’ for ubiquitination before being extracted by CHLORAD (Li *et al.*, 2022). Quantitative proteomic analyses identified additional intrachloroplast ubiquitinated proteins and their ubiquitination sites, further expanding the known role of the UPS and CHLORAD. These findings also revealed new potential targets, including proteins involved in photosynthesis, fatty acid biosynthesis, and plastid gene expression (Sun *et al.*, 2022). The accuracy of these findings has been questioned after a reanalysis of the raw proteomic data, which raised concerns about their reliability (van Wijk *et al.*, 2023; van Wijk and Adam, 2024). In response, additional experimental evidence has been presented in support of the original conclusions (Jarvis *et al.*, 2024), while ubiquitin-dependent degradation of organellar proteins has also been proposed in mitochondria (Liao *et al.*, 2020; Zhang *et al.*, 2022). Given the potential significance of this mechanism, a more in-depth investigation is required.

## Plastid precursor proteins as signalling source

When the cytosolic PQC/PPR machinery becomes overwhelmed by excessive precursor protein accumulation due to a complete disruption or inhibition of chloroplast differentiation, signalling mechanisms are activated. For example, lincomycin treatment blocks plastid translation, inhibiting the synthesis of at least two proteins involved in plastid import, the 1 MDa complex subunit Translocon Inner Membrane Complex 214 (Ycf1/Tic214) and a subunit of the 2 MDa heteromeric AAA-ATPase complex associated with the TIC complex, known as Ycf2, which functions as the import motor (Kikuchi *et al.*, 2013, 2018). In response, plastid-to-nucleus retrograde signalling pathways are activated, leading to the transcriptional down-regulation of *Photosynthesis-Associated Nuclear Genes* (*PhANGs*) (Fig. 1). When the lincomycin-induced down-regulation of *PhANGs* is ineffective, as observed in Arabidopsis seedlings lacking the GUN1 protein, protein translation inhibition occurs through disruption of plastid polyribosome assembly (Wu *et al.*, 2019b). This is followed by cytosolic detoxification mediated by the PPR pathway and the polyubiquitination of TOC34 translocon protein (Wu *et al.*, 2019a; Tadini *et al.*, 2020; Di Silvestre *et al.*, 2025). It has been suggested that the activation of PPR mechanisms in response to the overaccumulation of plastid precursor proteins plays a critical role in plastid-to-nucleus retrograde signalling (Wu *et al.*, 2019a). Accordingly, overexpressor lines of the cytosolic chaperone HSP90-1, which accumulates in response to lincomycin in *gun1* mutants, exhibit a genomes-uncoupled phenotype (Wu *et al.*, 2019a; Tadini *et al.*, 2020). In this context, PPR-derived cytosolic folding stress and the UPS probably play a major role in regulating the folding and stability of transcription factors that control *PhANG* transcription (Sugio *et al.*, 2009; Wu *et al.*, 2019a). Additionally, cytosolic HSP90-1 forms protein complexes with heat shock factors (HSFs). The HSF–HSP90 complex mediates cytosolic folding stress by releasing HSFs as HSP90 relocates to bind misfolded and damaged proteins. HSFs then trigger the up-regulation of the transcription factor gene *HSFA2*, which activates genes related to proteostasis (Nishizawa-Yokoi *et al.*, 2010). Therefore, the *HSFA2*-related retrograde signalling pathway integrates cytosolic folding stress and chloroplast-located proteotoxic folding stress independently of the GUN1-mediated retrograde signalling pathway (Fig. 1; Llamas *et al*., 2017; Jeran *et al*., 2021).

## Plastid protein trafficking and homeostasis

The chloroplast import machinery consists of two multimeric complexes: TOC and TIC. The TOC complex includes the pre-protein receptors TOC159 and TOC33, which expose their GTPase domains toward the cytosol to recognize cTPs of precursor proteins. Additional isoforms of these receptors exist in Arabidopsis, including TOC132, TOC120, TOC90, and TOC34 (Jarvis and López-Juez, 2013; Rochaix, 2022). TOC159 and TOC33 are primarily responsible for importing photosynthetic precursor proteins, whereas TOC34, TOC120, and TOC132 facilitate the import of housekeeping plastid precursors (Kessler and Schnell, 2009; Li and Chiu, 2010; Jarvis and López-Juez, 2013; Shi and Theg, 2013). TOC75 functions as the protein-conducting channel through which precursors traverse the outer membrane, and is shared by all TOC complexes (Jarvis and López-Juez, 2013).

While the composition of the TOC complex is well established, the organization of the TIC complex remains under debate. The ‘classic’ model proposes that TIC110, TIC40, and TIC20 form the core subunits, interacting with stromal chaperones such as CLPC/HSP93, cpHSC70, and HSP90C (Jarvis and López-Juez, 2013; Shi and Theg, 2013; Richardson *et al.*, 2017). More recently, an alternative 1 MDa TIC complex has been identified, comprising TIC56, TIC20, TIC100, and the plastid-encoded Tic214/Ycf1 (Kikuchi *et al.*, 2009, 2013; Nakai, 2018). Additionally, a 2 MDa complex, containing the plastid-encoded Ycf2 protein along with FTSH12 and FTSHi, has been discovered in Arabidopsis (Kikuchi *et al.*, 2018). Recent studies have characterized the structure of the 2 MDa complex in *Chlamydomonas reinhardtii* and *Pisum sativum*, highlighting its physical association with TOC–TIC components, particularly the subunits of the 1 MDa complex (Liang *et al.*, 2024a, b).

Proteins localized to the inner chloroplast envelope are imported via two distinct pathways: the stop–transfer pathway and the post-import pathway (Viana *et al.*, 2010). The post-import pathway, derived from the bacterial secretory system (cpSec2), enables full translocation of proteins into the stroma before membrane insertion. This pathway mediates the integration of specific inner membrane proteins, such as FTSH12 and TIC40 (Li *et al.*, 2017). Conversely, in the stop–transfer pathway, membrane proteins are arrested during translocation through the TIC complex and inserted laterally into the inner membrane, a key mechanism for handling hydrophobic proteins prone to aggregation (Viana *et al.*, 2010).

Chloroplast protein translocation requires energy, provided by import motors located on the stromal side of the machinery. Several chaperones, including cpHSP70, HSP90C, and HSP93, contribute to the TIC-associated motor (Sun and Jarvis, 2023). Two independent import motor complexes have been proposed: one involving HSP93 (CLPC) and TIC40, and the other comprising cpHSP70 proteins. The cpHSP70 complex acts as the primary import motor, as it tightly associates with the translocon and interacts with precursor proteins during import. However, HSP90C has also been found to be essential for protein translocation (Su and Li, 2010; Inoue *et al.*, 2013; Liu *et al.*, 2014). The CLPC chaperone, interacting with the CLPP proteolytic subunit, ensures PQC at the import site (Sjögren *et al.*, 2014; Flores-Pérez *et al.*, 2016). The recent resolution through cryo-EM of the 2 MDa complex has provided further insights into its association with the 1 MDa complex (Kikuchi *et al.*, 2018; Liang *et al.*, 2024a, b). In this model, the motor module is precisely positioned directly beneath TIC20, with this alignment facilitated by a peculiar, tilted angle. This conserved spatial arrangement of the 1 MDa and 2 MDa complexes enables efficient delivery of preproteins from TIC to the motor modules. The model strongly supports the role of the motor module as the primary motor for the import process through the 1 MDa TIC complex, although a complementary or regulatory role for the ‘classic’ import motor cannot be ruled out (Liang *et al.*, 2024a, b). Following import through the translocon machinery, plastid precursor proteins undergo maturation via proteolytic processing, where the transit peptide is cleaved by the stromal processing peptidase (SPP) (Sun *et al.*, 2021). Cleaved transit peptides are partially degraded by PREP oligopeptidases within the stroma. In Arabidopsis, two PREP paralogues, PREP1 and PREP2, exist, both dually targeted to chloroplasts and mitochondria that act on peptides ranging from 10 to 65 amino acids in length (Bhushan *et al.*, 2003, 2005; Moberg *et al.*, 2003; Ståhl *et al.*, 2005). Residual peptides of 8–23 amino acids undergo further degradation by OOP protease, also dual-located in chloroplasts and mitochondria (Kmiec *et al.*, 2013; Teixeira *et al.*, 2017). The coordinated activity of these enzymes generates short fragments of 3–7 amino acids, which are eventually broken down into single amino acids by M1 and M17–M20 aminopeptidases (Teixeira *et al.*, 2017; Kmiec *et al.*, 2018b).

Having reached the stromal compartment, proteins destined for the thylakoid membrane employ distinct insertion pathways, depending on their structure and targeting signals. Proteins with one or two transmembrane domains integrate into the thylakoid membrane via the spontaneous pathway, which requires no energy input (Celedon and Cline, 2013). In contrast, proteins with multiple transmembrane domains and large lumenal loops or tails require active insertion mechanisms. These rely on either the chloroplast signal recognition particle (cpSRP) pathway or the chloroplast-guided entry of tail-anchored proteins (cpGET) pathway (Celedon and Cline, 2013). The cpSRP pathway involves cpSRP54 (GTPase subunit), cpSRP43 (chaperone), LTD (stromal ankyrin protein), cpFTSY (membrane receptor), and Alb3 (integrase), and mediates the targeting and insertion of light-harvesting complex proteins (LHCPs). The cpSRP pathway, together with the chloroplast secretory translocase 1 (cpSEC1) pathway, also enables co-translational insertion of plastid-encoded proteins into the thylakoid membrane (Ouyang *et al.*, 2011; Ries *et al.*, 2020; Ji *et al.*, 2021; Sun and Jarvis, 2023). Meanwhile, the cpGET pathway directs the targeting of tail-anchored proteins, such as cpSEC1 components, via the ATPase GET3B (Anderson *et al.*, 2021). Lumenal proteins require additional translocation across the thylakoid membrane, occurring via either the cpSEC1 pathway or the chloroplast twin-arginine translocase (cpTAT) pathway, both of which depend on a lumenal targeting peptide of prokaryotic origin (Fig. 1; Albiniak *et al.*, 2012). While the cpSEC1 pathway utilizes the cpSECY1–cpSECE1 translocon and cpSECA1 ATPase motor to transport unfolded proteins, the cpTAT pathway enables proton motive force-driven transport of folded proteins via components cpTATA, cpTATB, and cpTATC, homologous to bacterial proteins (New *et al.*, 2018). The cpSec1 pathway also involves the chloroplast chaperonin CPN60, which assists in protein folding and stability (Endow *et al.*, 2015; Klasek *et al.*, 2020).

## Plastid protein folding and proteolysis

The plastid compartment contains a variety of proteases (72, both predicted and confirmed, as recently reviewed) that are essential for the generation and maintenance of a functional chloroplast proteome (van Wijk, 2024). These proteases perform several key functions, such as removing cTPs, cleaving the N-terminal methionine from plastid-encoded proteins, aiding in protein maturation, regulating protein turnover, and remodelling the chloroplast proteome in response to developmental or environmental signals (van Wijk, 2015). Plastid proteases can be classified into ATP-dependent and ATP-independent groups. The ATP-dependent category includes LON, CLP, and FTSH proteases, all of which belong to the larger AAA+ (ATP associated with various cellular activities) family. The ATP-independent category consists of DEG proteases (van Wijk, 2015; Nishimura *et al.*, 2016).

**Fig. 2.**
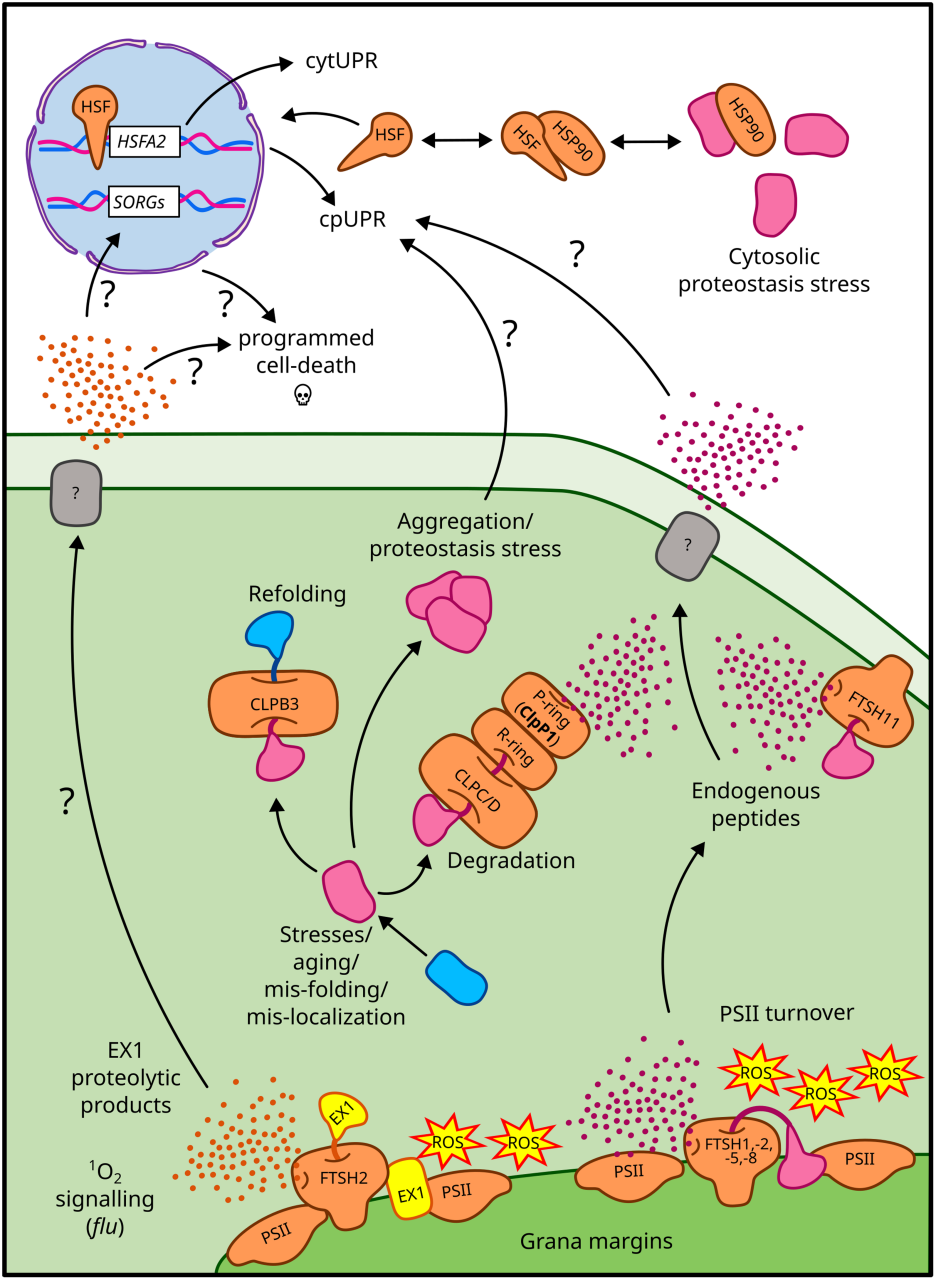
Plastid protein degradation and cpUPR. Protein misfolding, triggered by environmental stress, ageing, or mislocalization, leads to the formation of aggregates and activation of the chloroplast unfolded protein response (cpUPR). Protease complexes such as CLP and FTSH play a key role in degrading faulty proteins. Sites of reactive oxygen species (ROS) production are indicated. While studies in mitochondria suggest that peptides derived from protein degradation may function as signalling molecules, chloroplast peptide transporters have yet to be identified. Cytosolic heat shock factors (HSFs), which regulate the cytosolic unfolded protein response (cytUPR), also contribute to cpUPR activation. Functional proteins are shown in blue, while faulty proteins are depicted in pink. Plastid-encoded protein subunit ClpP1 is highlighted in bold, and signalling mediators, such as the retrograde signalling factor EX1, are marked in yellow. The EX1-mediated signalling pathway triggers the expression of singlet oxygen-responsive genes (SORGs) in the nucleus, eventually leading to programmed cell death.

The serine-type CLP (caseinolytic protease) machinery is the primary soluble stromal protease complex (Rodriguez-Concepcion *et al.*, 2019). This complex has a prokaryotic origin and is conserved across eukaryotes. In photosynthetic organisms, CLP proteases are found in both mitochondria and chloroplasts (van Wijk, 2015). The catalytically active P-ring consists of the CLPP3, P4, P5, and P6 subunits in a 1:2:3:1 ratio. The R-ring is made up of the plastid-encoded, catalytically active subunit ClpP1, along with the inactive subunits CLPR1, R2, R3, and R4, in a 3:1:1:1:1 ratio (Olinares *et al.*, 2011a, b; van Wijk, 2015). The core complex is stabilized by the small T1 and T2 subunits (Kim *et al.*, 2015). Protein substrates are delivered to the catalytic chamber of the CLP complex by specialized chaperone complexes, which form hexameric rings composed of CLPC1 and C2 subunits or CLPD subunits. These subunits feature a C-terminal protease-binding domain that enables them to interact with the CLP core complex (Fig. 2; Lee *et al.*, 2007; van Wijk, 2015). Additionally, CLPS1 and CLPF act as adaptors, recognizing and delivering substrates in collaboration with the CLPCs and CLPD chaperones. The chaperone rings also exhibit unfoldase activity, which helps linearize client proteins, facilitating their proteolysis in the core chamber (Nishimura *et al.*, 2013, 2015). Since CLPC chaperones are involved in protein import, they probably recruit the CLP protease core to the translocon complex, helping to remove altered proteins as they enter the chloroplast (Chou *et al.*, 2006; Sjögren *et al.*, 2014; Paila *et al.*, 2015; Flores-Pérez *et al.*, 2016). Although relatively few proteins have been directly confirmed as CLP substrates, many of these are involved in chloroplast metabolic pathways and housekeeping functions, such as RNA and protein synthesis and maturation. Nevertheless, this major proteolytic complex plays a pivotal role in maintaining chloroplast proteostasis (Stanne *et al.*, 2009; Rodriguez-Concepcion *et al.*, 2019).

The FTSH (filamentation temperature sensitive) group includes several membrane-embedded metalloproteases of prokaryotic origin that are conserved in eukaryotes and found in both mitochondria and chloroplasts. FTSH proteases have one or two transmembrane N-terminal domains, a central ATPase domain, and a C-terminal catalytic domain that coordinates a zinc ion. The catalytic chamber is formed by FTSH proteases associating into homo- or heterohexamers (van Wijk, 2015). In the Arabidopsis genome, there are 12 *FTSH* genes, nine of which encode proteins targeted to the chloroplast (Wagner *et al.*, 2012; van Wijk, 2015; Nishimura *et al.*, 2016). FTSH1, -2, -5, and -8 are localized to the thylakoid membranes, where they form heterohexamers consisting of different, non-functionally redundant subunits: Type A (FTSH1 or FTSH5) and Type B (FTSH2 or FTSH8), assembled in a 2:4 ratio of Type A to Type B subunits. Among these, FTSH5 and FTSH2 play the major roles, as they are the most abundant subunits (Sakamoto *et al.*, 2002, 2003). FTSH6 is also embedded in the thylakoid membranes and forms a complex with the heat shock protein HSP21 (Sedaghatmehr *et al.*, 2016). FTSH11, -7, -9, and -12 are localized to the inner envelope (van Wijk, 2015; Adam *et al.*, 2019). Five additional FTSH genes are present in the Arabidopsis genome, but the encoded proteins lack the zinc-binding motif, making them proteolytically inactive and known as FTSHi. Despite this, FTSHi proteins exhibit chaperone functions, as evidenced by similar findings in an *ftsh2* allele with a mutation in the zinc-binding motif (Wagner *et al.*, 2012; van Wijk, 2015; Nishimura *et al.*, 2016; Mishra and Funk, 2021). Although all FTSH proteases direct their catalytic chambers toward the stroma, they contribute to chloroplast homeostasis in diverse ways, interacting with different targets and partners (Sakamoto *et al.*, 2003; Kikuchi *et al.*, 2018; Adam *et al.*, 2019). The thylakoid-associated FTSH1, -2, -5, and -8 are directly involved in the turnover and assembly of PSII core subunits (Järvi *et al.*, 2016; Kato and Sakamoto, 2018). Additionally, FTSH11 plays an important role in Arabidopsis thermotolerance, as its absence severely reduces plant viability under heat stress (Chen *et al.*, 2006, 2018). Proteomic data also link this FTSH protease to housekeeping proteostasis functions (Adam *et al.*, 2019). FTSH12 has recently been shown to be part of the 2 MDa import machinery, alongside plastid-encoded Ycf2 and FTSHi proteins (Kikuchi *et al.*, 2018). While the function of FTSH6 remains poorly understood, it is thought to be involved in the regulation of thermomemory, high-light acclimation, and/or senescence-related processes (Żelisko *et al.*, 2005; Sedaghatmehr *et al.*, 2016). The functions of FTSH7 and FTSH9 remain unclear but may overlap, as these proteases appear to form heterocomplexes (Wagner *et al.*, 2012).

LON (LONg form) proteases are serine proteases conserved across all domains of life but were lost in cyanobacteria over evolutionary time. LON proteases can be either soluble (A-type) or membrane-anchored (B-type), with the latter found exclusively in Archaea (Rigas *et al.*, 2014). They have a long N-terminal domain, which, along with the central ATPase domain, selectively recognizes target proteins, and a C-terminal proteolytic domain (Botos *et al.*, 2004). Except in yeast cells, where LON forms heptamers, LON proteins generally assemble into hexameric rings to function properly, recognizing unstructured protein regions enriched in hydrophobic and aromatic residues (van Wijk, 2015). In Arabidopsis, there are four LON proteins, which are found in peroxisomes, mitochondria, and plastids. LON4 is the only isoform targeted to both chloroplasts and mitochondria, and has been associated with thylakoid membranes, although its function remains unknown (Ostersetzer *et al.*, 2007). Based on proteomic data, the Arabidopsis genome encodes four additional LON proteins that lack the catalytic domains; at least three of these are localized to chloroplasts (van Wijk, 2015).

Finally, DEG proteases are ATP-independent serine endopeptidases with an N-terminal proteolytic domain and one or two C-terminal PDZ protein–protein interaction domains. DEG proteases are peripherally associated with membranes but can also be found in soluble compartments (van Wijk, 2015). The proteolytic domain facilitates the formation of a native trimeric ring, which dimerizes via the PDZ domain to create the catalytic chamber. The function of DEG proteases is influenced by temperature and oligomerization state, and they play important roles in proteome maintenance, stress resolution, and apoptosis (Hansen and Hilgenfeld, 2013). The Arabidopsis genome encodes 16 DEG proteases, which are localized in various cellular compartments, including peroxisomes, the nucleus, mitochondria, and chloroplasts (Helm *et al.*, 2007; Schuhmann and Adamska, 2012; Schuhmann *et al.*, 2012; Dolze *et al.*, 2013; Basak *et al.*, 2014). Plastid-localized DEG1, -5, and -8 are located on the lumenal side of the thylakoid membrane, while DEG2, -7, and -11 are found in the stroma (van Wijk, 2015; Butenko *et al.*, 2018). The combined actions of stromal and lumenal DEGs are mainly responsible for the biogenesis and turnover of PSII. DEG1 forms a homohexamer, while DEG5 and DEG8 form a heterohexamer. Both complexes contribute to photosynthetic protein degradation during photoinhibition and are only partially redundant (Butenko *et al.*, 2018). Additionally, DEG1 works with FTSH and DEG2 to degrade D1 protein during photoinhibition repair by cleaving lumen-exposed regions (Kapri-Pardes *et al.*, 2007; Schuhmann and Adamska, 2012; van Wijk, 2015). In contrast to CLP proteases, DEG activity is influenced by environmental factors, such as pH for lumenal DEGs. DEG1 switches from an inactive monomer to an active hexamer at acidic pH (Kley *et al.*, 2011). This ensures their activation during daylight, when thylakoid lumen acidification occurs and photosynthetic proteins sustain damage. Moreover, DEG1 was recently found to be involved in lumenal pH-dependent non-photochemical quenching (NPQ) activation, by modulating the accumulation of violaxanthin de-epoxidase and PsbS (Aviv‐Sharon *et al.*, 2025).

## Chloroplast unfolded protein response(s)

Alterations or disruptions in proteolytic and protein folding/refolding processes, whether due to genetic mutations or to environmental fluctuations, can lead to the accumulation of damaged, unfolded, or misfolded proteins. These proteins persist as polypeptides with altered stoichiometry, localization, or temporal dynamics, and have a tendency to aggregate (Juszkiewicz and Hegde, 2018; Hegde and Zavodszky, 2019; Kong *et al.*, 2021). In the unicellular green alga *C. reinhardtii*, depletion of the ClpP1 subunit via a repressible chloroplast gene expression system activates plastid PQC mechanisms, defining the chloroplast unfolded protein response (cpUPR) (Ramundo *et al.*, 2014). ClpP1 depletion results in stalled cell proliferation and impaired photosynthetic performance, leading to repression of nuclear genes associated with photosynthesis. Conversely, nuclear-encoded chloroplast chaperones and proteases exhibit robust transcriptional and protein-level up-regulation. Notably, a subset of transcripts displays divergent behaviour compared with their corresponding proteins, indicating a comprehensive post-transcriptional regulatory mechanism governing cpUPR genes (Ramundo *et al.*, 2014). Moreover, *Chlamydomonas* cells lacking ClpP1 develop highly vesiculated chloroplasts and accumulate ATG8, suggesting the activation of autophagy (Ramundo *et al.*, 2014; see subsequent sections for a comprehensive discussion).

In Arabidopsis, cpUPR activation is observed when the plastid PQC system is disrupted, particularly in mutants lacking FTSH or CLP proteins. These mutants show increased levels of chaperones, proteases, and ROS-detoxifying enzymes (Kim *et al.*, 2009, 2013; Adam *et al.*, 2019; Dogra *et al.*, 2019a). A strong link between plastid gene expression and cpUPR has been demonstrated in seedlings treated with lincomycin, which inhibits plastid translation and prevents ClpP1 synthesis (Llamas *et al.*, 2017). Lincomycin treatment correlates with increased accumulation of the plastid unfoldase CLPB3, a response similarly observed in mutants of plastid RNA processing factors or ribosomal proteins. Additionally, lincomycin exposure leads to increased protein aggregation in the chloroplast stroma (Llamas *et al.*, 2017). As mentioned, the thylakoid transmembrane protease FTSH2, the most prominent subunit of the FTSH1, -2, -5, -8 heterocomplex, plays a pivotal role in chloroplast proteostasis and signalling (Yu *et al.*, 2004; Dogra *et al.*, 2019a). FTSH2 inactivation compromises PSII integrity, resulting in the accumulation of damaged proteins and triggering a cpUPR-like response. Despite some differences, the chloroplast proteome of the *ftsh2* mutant closely resembles that of *clp* mutants (Kim *et al.*, 2013; Dogra *et al.*, 2019a). Furthermore, the inner envelope-localized FTSH11 protease physically interacts with components of the translocon complex, as well as with CPN60 and CLPB3 chaperones. Proteomic analyses suggest that FTSH11 plays a central role in plastid proteostasis. In the *ftsh11* mutant, elevated accumulation of CLP protease components, plastid-localized HSP70s, and CLPB3 indicates an active cpUPR under physiological conditions (Adam *et al.*, 2019). Collectively, these studies demonstrate that disruptions in plastid PQC machinery result in the accumulation of misfolded proteins and protein aggregates, which trigger retrograde signalling that modulates nuclear gene expression, leading to cpUPR activation (Fig. 2; Ramundo and Rochaix, 2014; Ramundo *et al.*, 2014; Llamas *et al.*, 2017). A key player in this response is HSFA2, a transcription factor that links chloroplast signals to nuclear responses. *HSFA2* is transcriptionally activated in response to proteostasis imbalances and cpUPR activation (Fig. 2; Llamas *et al.*, 2017; Dogra *et al.*, 2019a). It plays a crucial role in stress response, particularly under heat stress and cytosolic protein-folding stress conditions (Nishizawa *et al.*, 2006; Schramm *et al.*, 2006). HSFA2 directly regulates plastid-localized chaperones such as CLPB3 and HSP21 (Nishizawa *et al.*, 2006; Schramm *et al.*, 2006). Its expression pattern peaks before that of plastid-localized stress-responsive factors following lincomycin treatment, mirroring—but to a lesser extent—the heat shock response (Llamas *et al.*, 2017). Importantly, *HSFA2* induction by lincomycin is suppressed by the chemical chaperone glycine betaine, further supporting its role in responding to misfolded or aggregated chloroplast proteins (Llamas *et al.*, 2017). Despite its crucial function, the mechanism by which cytosolic HSFA2 detects chloroplast protein misfolding remains unclear. In *Chlamydomonas*, the closest homologue to HSFA2, HSF1, is sequestered by Hsp90, which is inhibited by a GUN5-dependent tetrapyrrole metabolite, suggesting a potential signalling pathway (Kindgren *et al.*, 2012; Schmollinger *et al.*, 2013). However, in Arabidopsis, the *gun5* mutant does not exhibit altered CLPB3 expression following lincomycin treatment, indicating divergence in the signalling mechanism (Llamas *et al.*, 2017).

Beyond protein aggregation, the cellular system also prevents the accumulation of proteins that are out of stoichiometry with their interacting partners. In *Chlamydomonas*, depletion of the nuclear-encoded small subunit of Rubisco (SSU) triggers the formation of a repressive complex consisting of an octamer of the large Rubisco subunit (LSU) and the chaperone RAF1. This complex inhibits LSU translation by acting on the 5′-untranslated region (UTR) of the LSU gene (Wietrzynski *et al.*, 2021). Plastid protein homeostasis can also be perturbed by the overaccumulation of plastid-localized proteins that challenge the plastid folding machinery. In Arabidopsis, overaccumulation of the plastid ribosomal subunit PRPL4 triggers cpUPR, overlapping with heat and oxidative stress responses. As a result, PRPL4 overaccumulation is well tolerated and does not impair chloroplast function. Furthermore, PRPL4 does not interfere with plastid protein import, as no PRPL4 precursor proteins are detected, whereas the cytosolic PPR machinery is up-regulated (Tadini *et al.*, 2023). Interestingly, targeting an aggregation-prone human huntingtin fragment with an extended polyQ sequence to the chloroplast does not lead to its aggregation, unlike in animal cells, indicating the efficiency of the chloroplast proteostasis network in handling defective proteins (Llamas *et al.*, 2023). Nonetheless, the consequences of imbalanced protein synthesis and mislocalized proteins in chloroplasts, along with the mechanisms to prevent their accumulation, remain poorly understood.

## Plastid protein degradation as a signalling pathway

In various organisms, including Chlamydomonas and Arabidopsis, impairment of plastid-localized CLP or FTSH proteases has been shown to trigger a UPR-like signalling cascade in chloroplasts (cpUPR) (Kim *et al.*, 2009, 2013; Ramundo *et al.*, 2014; Llamas *et al.*, 2017; Adam *et al.*, 2019; Dogra *et al.*, 2019a). While the triggering factors and downstream responses are well characterized, the protein transducers and signalling molecules that physically link the plastid compartment to the nucleus remain unidentified (Fig. 2).

To bridge this gap, insights from mitochondrial biology provide valuable parallels. In *Caenorhabditis elegans*, the mitochondrial UR (mtUPR) is triggered by disruptions in mitochondrial proteostasis, such as impairment of the CLPX protease complex, homologous to the chloroplast CLP (Yoneda *et al.*, 2004; Benedetti *et al.*, 2006; Haynes *et al.*, 2007). This signalling pathway is thought to be mediated by small peptides derived from mitochondrial protein degradation. The mitochondrial inner membrane protein HAF-1 (Heme-responsive ABC transporter Family protein-1) was shown to export peptides (<20 amino acids) able to regulate nuclear gene expression (Haynes *et al.*, 2010). Peptide extrusion modulates ATFS-1 (Activating Transcription Factor associated with Stress-1), a dual-localized mitochondrial/nuclear transcription factor (Haynes *et al.*, 2010; Nargund *et al.*, 2012). Under optimal conditions, ATFS-1 is imported into mitochondria and degraded by LON protease. During mitochondrial stress, ATFS-1 is redirected to the nucleus to activate protective gene expression (Nargund *et al.*, 2012; Shpilka and Haynes, 2018). Inactivation of HAF-1 or ClpP-1 prevents ATFS-1 nuclear translocation and mtUPR activation (Haynes *et al.*, 2010). HAF-1 is the orthologue of MDL1 in *Saccharomyces cerevisiae*, responsible for exporting peptides from mitochondria during heat stress and regulating the respiratory chain (Young *et al.*, 2001; Augustin *et al.*, 2005; Arnold *et al.*, 2006).

A potential peptide-mediated signalling pathway has also been described in plants, in the Arabidopsis conditional lethal *flu* mutant (Dogra *et al.*, 2017). The FLU protein negatively regulates the Mg branch of tetrapyrrole biosynthesis. In the dark, *flu* mutants accumulate protochlorophyllide (Pchlide), which upon light exposure transfers energy to molecular oxygen, generating highly reactive singlet oxygen (^1^O_2_) (Meskauskiene *et al.*, 2001; op den Camp *et al.*, 2003). Rather than ^1^O_2_ accumulation itself, the lethal phenotype of *flu* mutants is attributed to programmed cell death mediated by selective degradation of the EXECUTER1 (EX1) protein within chloroplasts (Fig. 2). EX1 has been localized to the grana margins, specifically where chlorophyll synthesis occurs and where PSII is subjected to turnover. In grana margins, EX1 interacts with PSII subunits undergoing maintenance, together with the FTSH repair machinery and proteins involved in chlorophyll synthesis. Free tetrapyrroles and chlorophylls released during PSII repair generate ^1^O_2_ (Wang *et al.*, 2016). EX1 acts as a ^1^O_2_ detector through its Trp643 residue in the singlet oxygen sensor domain. Oxidation of Trp643 triggers EX1 degradation, activating singlet oxygen-responsive genes (SORGs) and inducing programmed cell death (Dogra *et al.*, 2019b). Both EX1 degradation and *flu*-dependent lethality are prevented by inactivation of FTSH2, linking chloroplast protein degradation and peptide generation with signalling, similar to mitochondria (Dogra *et al.*, 2017). As discussed, chloroplast proteins undergo multiple quality control mechanisms throughout their lifetime, under both normal conditions and stress. Proteome maintenance and remodelling involve proteolysis, generating peptides as by-products (Fig. 2). In *Physcomitrella patens*, the analysis of the chloroplast peptidome revealed the presence of peptides derived from photosynthesis-related proteins (Fesenko *et al.*, 2015). Intriguingly, some antimicrobial peptides were identified among the degradation products of chloroplast proteins following methyl jasmonate treatment (Khazigaleeva *et al.*, 2017; Ramada *et al.*, 2017; Fesenko *et al.*, 2019). Furthermore, Arabidopsis mutants lacking the organellar oligopeptidases PREP1/-2 and OOP accumulate plastid-derived peptides. In these mutants, 180 peptides from 95 proteins, ranging from six to 30 amino acids in length, were identified. These mutants exhibit reduced growth and photosynthesis, leading to lower reproductive fitness, while simultaneously up-regulating protective genes, particularly those involved in biotic stress responses. These findings suggest that the accumulation of chloroplast-derived peptides serves as a stress-responsive signalling mechanism (Kmiec *et al.*, 2018*a*).

Notably, peptide-based signalling in eukaryotes show analogies with bacterial quorum-sensing mechanisms. In bacteria, quorum-sensing peptides (QSPs) mediate cell–cell communication, coordinating collective behaviours. Some QSPs can induce cell death in competing bacteria, acting as natural antibiotics (Kumar *et al.*, 2013; Kumar and Engelberg-Kulka, 2014). This functional parallelism highlights the possibility that peptides may act as signalling molecules, coordinating stress responses across various cellular compartments in eukaryotic cells. However, since no direct experimental evidence of peptide-mediated chloroplast signalling has been provided yet, further dedicated studies are needed to elucidate this process.

## Chloroplast quality control and degradation mechanisms

Beyond the well-characterized senescence-driven degradation mechanisms, required for redistribution of nutrients during ageing and seed production, chloroplast degradation pathways function to prevent damaged chloroplasts, whether compromised by endogenous or exogenous stressors, from harming the cellular environment (Woodson, 2022). Chloroplast degradation is governed by a complex network of signalling pathways, many of which remain only partially understood. This process can occur through multiple pathways, often involving autophagy (Xie *et al.*, 2015; Zhuang and Jiang, 2019; Qi *et al.*, 2021). Autophagy is a highly conserved and dynamic process in eukaryotes that facilitates the degradation and recycling of cytoplasmic components and organelles. In response to environmental and developmental cues, cellular material is delivered to the lysosome in animal cells or the central vacuole in yeast and plant cells, ensuring macromolecule turnover and cellular homeostasis (van Wijk, 2015; Marshall and Vierstra, 2018; Qi *et al.*, 2021). Autophagy is further categorized into macroautophagy and microautophagy (Marshall and Vierstra, 2018; Zhuang and Jiang, 2019). Studies in *S. cerevisiae* have identified >40 autophagy-related genes (*ATG* genes) shared between macro- and microautophagy (Mizushima *et al.*, 1998; Ichimura *et al.*, 2000; Nakatogawa *et al.*, 2009). These early studies paved the way to unravel the autophagic components conserved in plants (Marshall and Vierstra, 2018; Yoshimoto and Ohsumi, 2018). Among ATG proteins, the ubiquitin-like ATG8 serves as a key marker of the autophagosome membrane. It plays a crucial role in autophagosome formation, cargo recognition, transport to the vacuole or lysosome, and membrane fusion (Hansen and Johansen, 2011; Wesselborg and Stork, 2015; Nieto-Torres *et al.*, 2021). Macroautophagy consists of the complete encapsulation of cytoplasmic portions and/or organelles within an autophagosome, a double membrane vesicle mainly formed from the endoplasmic reticulum (ER) in plants, which is subsequently delivered to the vacuole for degradation (Bassham, 2009; Le Bars *et al.*, 2014; Zhuang *et al.*, 2017; Marshall and Vierstra, 2018; Zhuang and Jiang, 2019; Woodson, 2022). In contrast, during microautophagy, cellular components near the vacuole or lysosome are directly engulfed through membrane invagination, either independently or in conjunction with autophagosome formation (Mijaljica *et al.*, 2011; Oku *et al.*, 2017; Zhuang and Jiang, 2019; Woodson, 2022). When entire chloroplasts are dismantled in the vacuole, delivered by either macro- or microautophagic mechanisms, the process is broadly indicated as chlorophagy (Woodson, 2022). Analysis of dark-incubated Arabidopsis leaves revealed that, although senescence was triggered in the autophagy-deficient *atg4a4b-1* mutant, it failed to reduce the number and size of chloroplasts. This finding links senescence to autophagic processes that specifically target chloroplasts through macro- or microautophagy (Yoshimoto *et al.*, 2004; Wada *et al.*, 2009). Furthermore, UV-damaged chloroplasts were observed to be surrounded by autophagosomes and later delivered to the vacuole through macroautophagy. Accordingly, autophagy plays a crucial role in the response to UV-B irradiation, as *atg* mutants exhibit increased sensitivity to treatment and fail to remove damaged chloroplasts (Izumi *et al.*, 2017). High-light treatment was also found to be associated with chlorophagy, which was later characterized as an autophagosome-dependent microautophagy process, since chloroplasts partially marked by the autophagosome were engulfed by the vacuole (Izumi *et al.*, 2017; Nakamura *et al.*, 2018).

Autophagosome-dependent chloroplast degradation can also proceed gradually through specialized vesicles, each targeting specific components (Woodson, 2022). The ATG8-Interacting Protein 1 (ATI1) facilitates vesicular trafficking from the ER and chloroplasts to the vacuole (Honig *et al.*, 2012). Plastid-derived vesicles, known as ATI1-plastid associated bodies (ATI1-PS), containing ATI1, thylakoid proteins, and other chloroplast components, have been observed under carbon starvation (Honig *et al.*, 2012; Michaeli *et al.*, 2014). Similarly, small Rubisco-containing bodies (RCBs) transport Rubisco and stromal proteins to the vacuole for resource recycling under senescence and carbon starvation conditions (Chiba *et al.*, 2003; Izumi *et al.*, 2010; Izumi and Ishida, 2011). Additionally, small starch granule-like (SSGL) structures facilitate starch granule degradation, contributing to diurnal starch metabolism (Wada *et al.*, 2009; Wang *et al.*, 2013). Chloroplast degradation can also occur independently of autophagosomes via multiple mechanisms (Woodson, 2022). Senescence-associated vesicles (SAVs), for example, are single-membrane structures containing stromal and thylakoid proteins, along with proteases such as SAG12, which exhibit strong proteolytic activity (Otegui *et al.*, 2005). Under abiotic stress, however, a different vesicle-mediated, autophagy-independent chloroplast degradation pathway is activated (Wang and Blumwald, 2014). The thylakoid-localized chloroplast vesiculation (CV) protein interacts with PSBO and other photosystem proteins, destabilizing them and forming CV-containing vesicles (CCVs) that transport chloroplast proteins to the vacuole. However, the molecular mechanisms of this process remain quite elusive (Wang and Blumwald, 2014; Woodson, 2022). Independent of autophagosome formation, a selective, ubiquitin-dependent chloroplast degradation pathway has also been identified (Woodson *et al.*, 2015; Lemke *et al.*, 2021; Fisher *et al.*, 2022). In Arabidopsis, the *fc2* mutant, which lacks plastid ferrochelatase 2, accumulates protoporphyrin IX (Proto IX) instead of converting it to haem. Upon light exposure, Proto IX generates singlet oxygen, causing chloroplast degradation and cell death under diurnal light cycles (Scharfenberg *et al.*, 2015; Woodson *et al.*, 2015). Under continuous light, degrading chloroplasts adhere to the vacuole and release materials via large vesicles, resembling a fission-type microautophagy (Lemke *et al.*, 2021; Fisher *et al.*, 2022). This process requires the PUB4 E3 ubiquitin ligase for targeted disassembly of ROS-damaged chloroplasts (Woodson *et al.*, 2015).

**Fig. 3.**
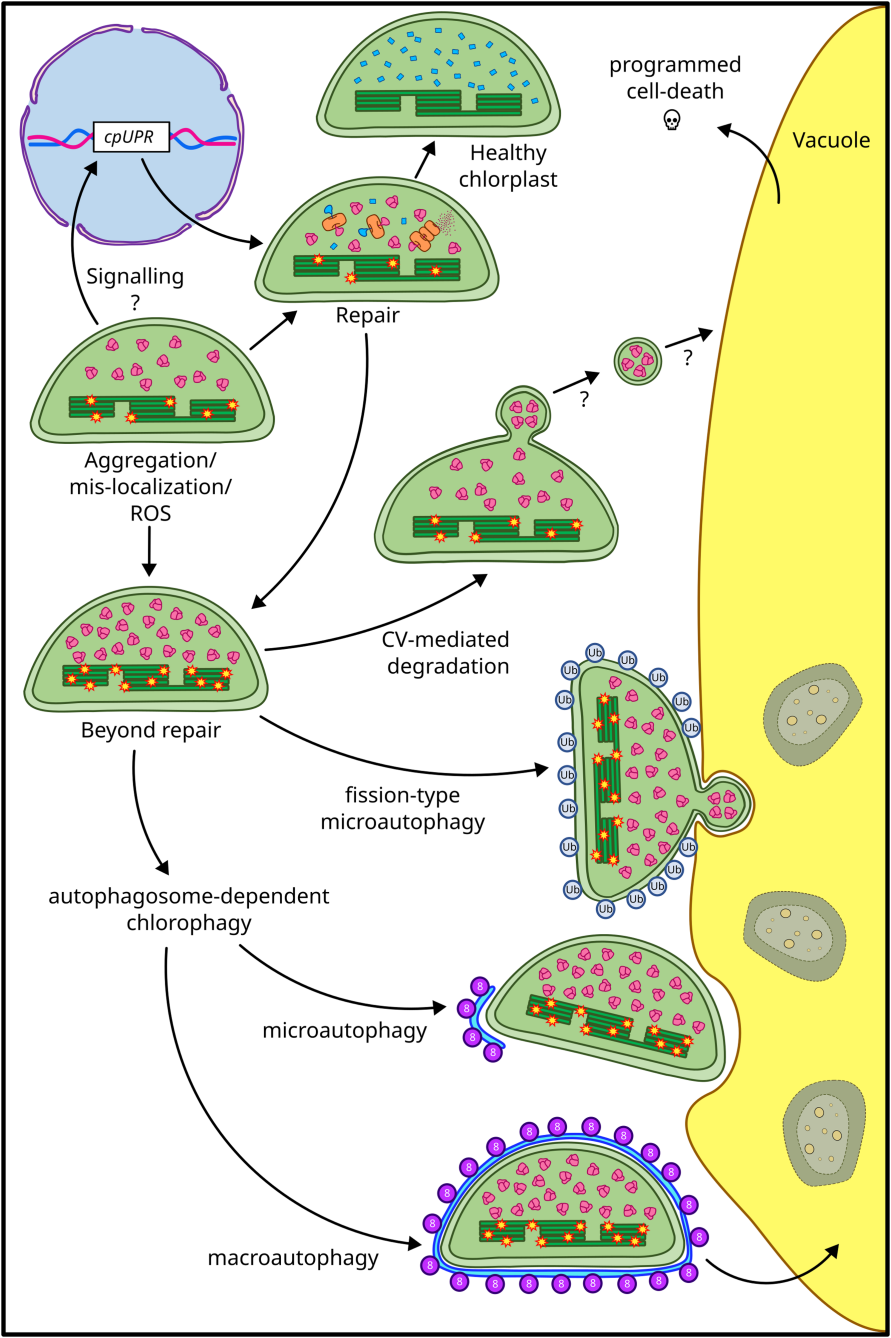
Chloroplast quality control and degradation pathways. Accumulation of damaged proteins within chloroplasts triggers the nuclear expression of cpUPR-related genes, encoding plastid-targeted chaperones and proteases, in an attempt to restore chloroplast proteostasis. However, the prolonged presence of faulty proteins and aggregates, exacerbated by ROS and other stressors, activates chloroplast-dismantling mechanisms. These include whole-chloroplast autophagosome-mediated degradation (chlorophagy), chloroplast vesiculation-mediated pathways (CV), or fission-type microautophagy, similar to those observed in mitochondria. Ubiquitin (light blue circles, Ub) and ATG8-dependent pathways (ATG8 purple circles; autophagosome depicted in blue) play crucial roles in marking chloroplasts for degradation. Ultimately, the accumulation of damaged chloroplasts within the cell leads to vacuole-mediated programmed cell death.

When plastids sustain mild damage due to disruptions in protein homeostasis and aggregation, repair pathways such as the cpUPR are activated. However, prolonged stress or irreversible plastid dysfunction triggers chloroplast dismantling (Fig. 3; Liu and Bassham, 2012; Llamas and Pulido, 2022; Woodson, 2022). The interplay between cpUPR and chloroplast dismantling has been observed in *Chlamydomonas*, where depletion of the ClpP1 protease subunit results in highly vesiculated chloroplasts and the activation of autophagy (Ramundo *et al.*, 2014). In Arabidopsis, the *gun1 fsth2* and *gun1 ftsh5* double mutants, which exhibit defects in PQC and retrograde signalling, activate PUB4-mediated chloroplast degradation (Tadini *et al.*, 2020; Jeran *et al.*, 2021). Moreover, enhanced chlorophagy was observed in *ftsh2* and *ftsh5* mutants following high-light exposure, suggesting that PSII damage can initiate chloroplast dismantling through autophagic mechanisms (Nakamura *et al.*, 2018). Additionally, extensive ubiquitination of impaired proteasomes induces ATG8-mediated removal via proteaphagy (Marshall *et al.*, 2015). Intriguingly, proteasomes also degrade autophagy-initiating factors (Qi *et al.*, 2020). These degradation pathways are deeply interconnected, requiring further study to fully elucidate their regulation and specificity (Raffeiner *et al.*, 2023; Yu and Hua, 2023; Sharma *et al.*, 2024).

## Protein mislocalization and misaccumulation as a signal for plastid degradation

The accumulation of stoichiometrically unbalanced protein subunits, known as orphan proteins, disrupts both cellular and plastid homeostasis. Similarly, protein mislocalization to incorrect cellular compartments exposes these proteins to environments lacking their interaction partners or retaining cleavable transit peptides. In both scenarios, exposed hydrophobic regions resulting from improper interactions or cleavage increase the risk of protein aggregation (Juszkiewicz and Hegde, 2018). To mitigate potential functional disruptions, cells often initiate targeted degradation of orphan proteins (Fig. 3). For instance, attempts to overexpress the plastid ribosomal subunit S1 (PRPS1) lead to its degradation by the stromal CLP protease complex (Yu *et al.*, 2012; Tadini *et al.*, 2023). PRPS1 is an atypical ribosomal subunit associated with the small ribosomal subunit and also functions as an mRNA-bound translation initiation factor (Merendino *et al.*, 2003). Overexpression of *PRPS1* impairs plastid translation, induces a chlorotic/virescent phenotype, activates both micro- and macroautophagy pathways for plastid degradation, and promotes early flowering (Tadini *et al.*, 2023). Notably, PRPS1 degradation is linked to the down-regulation of its cytosolic mRNA, a process that depends on PRPS1 protein degradation since it does not occur when proteolysis is inhibited. This highlights a tight interplay between plastid protein degradation and cytosolic post-transcriptional regulation, suggesting communication pathways mediated by proteolytic products (Tadini *et al.*, 2023).

The thylakoid lumen protein PSBO1 has recently emerged as a key regulator of chloroplast homeostasis and degradation. While its primary role is in the oxygen evolving complex (OEC), facilitating water splitting to regenerate the oxidized P680^+^ after charge separation (Ifuku *et al.*, 2010), accumulating evidence suggests additional functions. PSBO1 levels correlate with chloroplast degradation, as *psbo1* knockout lines can rescue the lincomycin sensitivity of *gun1* seedlings, in which thylakoid formation is impaired, suggesting a role in plastid quality control (Di Silvestre *et al.*, 2025). Additionally, PSBO1 mislocalization to the stroma via green fluorescent prtoein (GFP) tagging has been linked to protein degradation and a chlorotic phenotype (Jiang *et al.*, 2017). However, artificially mistargeted PSBO in isolated chloroplasts is efficiently degraded by the CLP complex, indicating that stromal PSBO accumulation can be at least partially managed by the plastid proteostasis system (Halperin and Adam, 1996). On the other hand, PSBO1–GFP-expressing plants exhibit chloroplast dismantling and programmed cell death, leading to a variegated phenotype that can be rescued by reducing *CV* expression (Di Silvestre *et al.*, 2025). PSBO interacts with CV in vesicle structures budding from chloroplasts to the vacuole, a hallmark of chloroplast degradation and senescence-driven degradation (Wang and Blumwald, 2014). Additionally, the vacuole-localized papain-like cysteine protease RD21A, a key player in vacuole-mediated programmed cell death, targets PSBO in *Chlamydomonas* (van Midden *et al.*, 2024). Interestingly, removing PSBO1 and other OEC proteins alleviates the conditional lethality of *proton gradient regulation 5* (*pgr5*) mutants under fluctuating light conditions (Suorsa *et al.*, 2016). While *pgr5* lethality in fluctuating light has not been explicitly linked to cell death, it is likely to share similarities with *flu* and *fc2* mutants grown under dark/light cycles (Meskauskiene *et al.*, 2001; op den Camp *et al.*, 2003; Woodson *et al.*, 2015). Mislocalized PSBO1–GFP proteins form large structures incorporated into CV-induced budding vesicles, possibly due to the formation of multimeric structures or aggregates targeted for vacuolar clearance (Di Silvestre *et al.*, 2025). In line with this, overexpression of the stromal chaperone *HSP90C* rescues the chlorotic phenotype caused by PSBO1–GFP mislocalization, suggesting that chaperone-mediated degradation mechanisms are activated and implying aggregate formation (Jiang *et al.*, 2017). Although protein aggregate vesiculation is not well described in chloroplasts, mitochondria are known to eliminate damaged material through asymmetric division. This process helps compartmentalize protein aggregates and initiate the disassembly of the affected portion, thereby reducing proteotoxic stress (Rujano *et al.*, 2006; Melentijevic *et al.*, 2017; Lyamzaev *et al.*, 2022).

In this context, PSBO1 mislocalization to the stroma probably promotes aggregation due to its intrinsically disordered lumenal transit peptide, rather than simply its presence. These aggregates may be inaccessible to the soluble CLP protease complex, preventing their degradation and triggering chloroplast vesiculation. In extreme cases, this may activate cell death pathways (Fig. 3). The role of PSBO1 in plastid proteostasis is further supported by its unique accumulation pattern. Alongside its homologue PSBO2, PSBO1 accumulates in non-photosynthetic plastids out of stoichiometric balance with other OEC subunits and in the absence of most photosynthesis-related proteins, as observed in spectinomycin- and lincomycin-treated seedlings (Köhler *et al.*, 2016; Tadini *et al.*, 2020; Di Silvestre *et al.*, 2025). Moreover, PSBO1 accumulation is detected in plastids lacking the canonical photosynthesis-related TOC/TIC import machinery, as seen in *tic56* and *ppi2* Arabidopsis mutants (Köhler *et al.*, 2016), suggesting that PSBO is imported through a general housekeeping pathway rather than the dedicated photosynthetic import route.

## Conclusions and perspectives

This review summarizes the mechanisms that regulate the intracellular trafficking of nuclear-encoded proteins targeted to plastids, with a particular focus on the key pathways triggered by the accumulation of mislocalized or orphan proteins. Such proteins, when present in excess or out of stoichiometric balance, expose hydrophobic regions and intrinsically disordered transit peptides, making them prone to aggregation. Protein aggregates pose a significant threat to cellular homeostasis, and distinct quality control mechanisms operate within different compartments to prevent or resolve their accumulation. Mislocalized plastid precursor proteins in the cytosol are typically recognized by the UPS and degraded (Fig. 1), whereas plastids rely on chaperones, proteases, and the activation of the cpUPR to manage protein aggregation (Fig. 2).

In mitochondria, degradation products such as peptides serve as signalling molecules that coordinate organelle homeostasis; however, analogous pathways in chloroplasts remain poorly understood and largely speculative (Fig. 2). Identifying plastid-localized peptide transporters and their associated transcription factors will be crucial for uncovering potential plastid-to-nucleus communication pathways. Interestingly, the mislocalization of specific proteins may itself serve as a signal for proper plastid compartment development. Within the framework of peptide-mediated signalling, mislocalized proteins may be targeted by specialized proteases, generating distinct degradation patterns that act as regulatory cues for intracellular protein trafficking and homeostasis. Alternatively, plastid protein aggregates are managed through the activation of vesiculation and dismantling mechanisms, preventing damaged chloroplasts from producing ROS and harming other cellular compartments (Fig. 3). In mitochondria, protein aggregates trigger vesiculation pathways, asymmetric division, and mitophagy to remove insoluble deposits. While similar processes in chloroplasts are less well characterized, the atypical photosynthesis-related protein PSBO1 has been observed forming large clumps within vesicles budding from chloroplasts. Mislocalization of PSBO1 to the stroma activates chlorophagy and programmed cell death. Notably, PSBO1 exhibits unique characteristics: (i) it is imported via a non-photosynthetic pathway; (ii) it accumulates in plastids that lack photosynthetic machinery; and (iii) it plays a direct role in chloroplast degradation. These features suggest that PSBO1 serves as a key regulatory protein, linking proplastid-to-chloroplast differentiation with plastid quality control.

In this context, PSBO1 may function as a pioneer protein, reaching the plastid compartment during early developmental stages. When correctly translocated to the thylakoid lumen, it facilitates chloroplast differentiation; however, if mislocalized, it may signal plastid dysfunction and initiate plastid dismantling or, in severe cases, cell-wide degradation processes.
